# Its Claims and Advantages

**Published:** 1917-09-08

**Authors:** 


### THE ROYAL NAVAL MEDICAL SERVICE.

### Its Claims and Advantages.

Under the Military Service Act it has become compulsory for every medical practitioner of military age and a duty for those above that age up to forty-five to enrol for service in the Navy or Army.

It is not the purpose of this article to plead the claim of the Naval Medical Service against the Army, but only to indicate some of the considerations that may influence the choice of the individual when called upon for service. The Naval Service is exacting in its claims. The duties, except during and after contact with the enemies' forces, are monotonous rather than arduous. The health of the *personnel* has been excellent throughout the war, and there is generally little call for professional skill.

A veil has been wisely drawn to a large extent over the doings of the Fleets, but we are allowed to gather that there are long and dreary phases of patrol duty in the North Sea and that opportunities of landing are few.

But to a keen spirit the attractions of the life must be great. It is no small thing to feel that one belongs to the greatest and most efficient fighting machine that the world has ever produced, a machine that more than anything else is contributing to that final victory that we now feel is assured. And the surgeon is as much a part of that machine as anyone else. He shares its glories and he shares its risks.

We know that the toll of life among the medical officers of the Fleet has been heavy, we know also that their work has been done well and has been duly appreciated after each naval action. And if the life is frequently monotonous, let it be remembered that it is being shared with a body of men to whom the whole world gives ungrudging admiration. There is no better companionship than is to be met in a naval mess. Given a sound constitution, a cheery disposition, and immunity from sea-sickness, no man will regret his choice of the Navy.

One other essential is handiness. The naval surgeon should be a true "handy man," for he will have to adapt himself to conditions calling for much ingenuity in handling the wounded on board ship. Particulars as to pay, etc., are not in the scope of this article, but can be obtained on application to the Director-General, Medical Department, Admiralty, S.W.

During the war the usual peace-time regulations are suspended, though doubtless they will come into force again when conditions are normal. A brief *résumé* of them is given below.

